# A massively parallel nonoverlapping additive Schwarz method for discontinuous Galerkin discretization of elliptic problems

**DOI:** 10.1007/s00211-015-0718-5

**Published:** 2015-04-01

**Authors:** Maksymilian Dryja, Piotr Krzyżanowski

**Affiliations:** University of Warsaw, Warsaw, Poland

**Keywords:** 65N55

## Abstract

A second order elliptic problem with discontinuous coefficient in 2-D or 3-D is considered. The problem is discretized by a symmetric weighted interior penalty discontinuous Galerkin finite element method with nonmatching simplicial elements and piecewise linear functions. The resulting discrete problem is solved by a two-level additive Schwarz method with a relatively coarse grid and with local solves restricted to subdomains which can be as small as single element. In this way the method has a potential for a very high level of fine grained parallelism. Condition number estimate depending on the relative sizes of the underlying grids is provided. The rate of convergence of the method is independent of the jumps of the coefficient if its variation is moderate inside coarse grid substructures or on local solvers’ subdomain boundaries. Numerical experiments are reported which confirm theoretical results.

## Introduction

In this paper we consider a second order elliptic equation

$$\begin{aligned} -{\text {div}}(\rho \nabla u) = f, \end{aligned}$$

with homogeneous Dirichlet boundary condition, where the diffusion coefficient $\rho$ is a discontinuous function. The problem is discretized by a symmetric weighted interior penalty discontinuous Galerkin (DG) finite element method with simplicial nonmatching elements and piecewise linear functions. Our goal is to design and analyze a two-level nonoverlapping additive Schwarz method (ASM), see e.g. [16], for solving the resulting discrete problem with rate of convergence independent of the jumps of the coefficient and with a potential for a very high level of fine grained parallelism.

Usually, two level ASMs for discretizations on fine mesh of size $h$ are defined by introducing a partitioning of the domain into subdomains of size $H> h$, where local solvers are applied in parallel. A global coarse problem is then based on the same partitioning. This method has recently been generalized for nonoverlapping domain decomposition methods for DG discretizations, allowing the coarse grid with mesh size $\mathcal {H}\le H$ to be a refinement of the original partitioning into subdomains where local solvers are applied—see e.g. [1, 14].

In the present paper, we take a different approach and consider local solvers’ subdomains of size $H$ and a coarse grid of size $\mathcal {H}$ such that $\mathcal {H}\ge H$. Therefore, in an extreme case our local solver subdomain can be as small as a single element of the fine mesh (so then $H= h$); on the other extreme, it can be set equal to the coarse grid cell, $H= \mathcal {H}$, the usual approach. By allowing small subdomains we substantially increase the level of parallelism of the method. Very small and cheap to solve local systems come in huge quantities, which can be an advantage on new multi-threaded processors. Moreover, small subdomains give more flexibility in assigning them to processors for load balancing in coarse grain parallel processing. In this way, an additional level of domain partitioning gives the user more parameters to fine tune the actual parallel performance, and thus overall efficiency, of the preconditioner for a given hardware architecture. The increased level of parallelism affects the condition number of the preconditioned system in a controlled way which we prove is, roughly speaking, of order $O\left( {\mathcal {H}^2}/{h H}\right)$. Numerical experiments reported in this paper confirm the bound is sharp in terms of mesh sizes.

In particular, if $H= h$ we get the condition number of order $O(\mathcal {H}^2/h^2)$ which then gradually improves, as $H$ increases, to well-known $O(\mathcal {H}/h)$ when $H= \mathcal {H}$, see [14]. It is also not surprising that if all partitionings scale in the same way so that $\mathcal {H}/H$ and $H/h$ are kept constant, then the condition number remains bounded independently of the actual values of $\mathcal {H},H,h$.

The method discussed in the paper makes the preconditioned system condition number independent of the jumps of diffusion coefficient $\rho$, under the assumption that the distribution of the coefficient satisfies certain assumptions. We identify two such cases: when the variation of the coefficient is mild inside cells of the coarse grid, or when the coefficient is close to a constant on the skeleton of the partitioning into local solvers’ subdomains.

The ASM discussed here is a generalization of [11] and, for continuous coefficient, of [1, 14]. See also [3] for a similar concept for continuous FE discretization where functions of the coarse space were constant inside substructures on which the original region was partitioned. The authors are unaware of other results concerning the influence of the sizes of local solver subdomains and the coarse grid on the condition number when the former grid is a refinement of the latter, in the context of DG methods for the discretization of elliptic problems with discontinuous coefficient. Other recent works towards domain decomposition preconditioning of DG discretizations of problems with strongly varying coefficients include [2, 5, 6].

The paper is organized as follows. In Sect. 2, differential and discrete DG problems are formulated. In Sect. 3, ASM for solving the discrete problem is designed and analyzed. Numerical experiments are presented in Sect. 4.

For nonnegative scalars $x,y$, we shall write $x \lesssim y$ if there exists a positive constant $C$, independent of $x$, $y$ and mesh parameters $h,H,\mathcal {H}$, and of jumps of the diffusion coefficient $\rho$ as well, such that $x \le Cy$. If both $x \lesssim y$ and $y \lesssim x$, we shall write $x \simeq y$.

## Differential and discrete DG problems

Let $\Omega$ be a bounded open polyhedral domain in $R^d$, $d\in \{2,3\}$, with Lipschitz boundary $\partial \Omega$. We consider the following variational problem for given $f \in L^2(\Omega )$ and $\rho \in L^\infty (\Omega )$:

Find $u^* \in H^1_0(\Omega )$ such that

1 $$\begin{aligned} a(u^*,v) = (f,v)_\Omega , \quad \forall v \in H^1_0(\Omega ), \end{aligned}$$

where

$$\begin{aligned} a(u,v) = \int _\Omega \rho \,\nabla u \cdot \nabla v\, dx, \quad (f,v)_\Omega = \int _\Omega f v\, dx. \end{aligned}$$

We assume that there exist constants $\alpha _0$ and $\alpha _1$ such that $0 < \alpha _0 \le \rho \le \alpha _1$ a.e. in $\Omega$ so that (1) is well-posed. Without loss of generality we shall additionally suppose that $\alpha _0 \ge 1$, which can always be guaranteed by simple scaling. This assumption will simplify the proof in Sect. 3.

In what follows we will analyze a preconditioner for a system of algebraic equations arising from a discretization of (1) with DG finite element method. The corresponding finite element spaces and the discrete problem are introduced below in the following subsection.

### Finite element spaces and DG discretization

Let ${\mathcal {T}_{h}}$ denote an affine, shape-regular but not necessarily conforming partition of $\Omega$ by triangles in 2-D or tetrahedrons in 3-D, ${\mathcal {T}_{h}}= \{{K}_1,\ldots ,{K}_{N_h}\}$. We shall refer to ${\mathcal {T}_{h}}$ as the “fine mesh”. The diameter of element ${K}\in {\mathcal {T}_{h}}$ will be denoted by $h_{K}$ (assumed uniformly bounded from above by an absolute constant) and we collect all elements’ diameters in a multi-parameter $h=(h_{{K}_1},\ldots , h_{{K}_{N_h}})$.

By ${\mathcal {E}_{h}^{0}}$ we denote the set of all common (internal) faces (edges in 2-D) of elements in ${\mathcal {T}_{h}}$, so that $e \in {\mathcal {E}_{h}^{0}}$ iff $e = \partial {K}_i \cap \partial {K}_j$ is of positive measure. We will use symbol $\mathcal {E}_{h}$ to denote the set of all faces (edges in 2-D) of fine mesh ${\mathcal {T}_{h}}$, that is those either in ${\mathcal {E}_{h}^{0}}$ or on the boundary $\partial \Omega$. For $e\in \mathcal {E}_{h}$ we set $h_e = {\text {diam}}(e)$.

We assume that ${\mathcal {T}_{h}}$ is shape- and contact-regular in the sense of [9, Definition 1.38], that is, it admits a matching submesh $\mathcal {T}_{\tilde{h}}$ which is shape-regular and such that for any ${K}\in {\mathcal {T}_{h}}$ the ratios of $h_{K}$ to diameters of simplices in $\mathcal {T}_{\tilde{h}}$ covering ${K}$ are uniformly bounded by an absolute constant. In consequence, if $e = \partial {K}_i \cap \partial {K}_j \in {\mathcal {E}_{h}^{0}}$, then

2 $$\begin{aligned} h_{e} \simeq h_{{K}_i} \simeq h_{{K}_j}. \end{aligned}$$

Moreover, the number of neighboring elements in ${\mathcal {T}_{h}}$ is bounded by an absolute constant.

For $p\in \{0,1\}$, we denote by ${\mathcal {P}_{p}}$ the set of polynomials of degree not greater than $p$. Then we define the finite element space ${{V}_{h}}$ in which we will approximate (1),

3 $$\begin{aligned} {{V}_{h}}= \{v\in L^2(\Omega ): v_{|_{K}} \in {\mathcal {P}_{1}} \text { for all } {K}\in {\mathcal {T}_{h}}\}. \end{aligned}$$

Note that traces of functions from ${{V}_{h}}$ are multi-valued on the skeleton $\mathcal {E}_{h}$.

We discretize (1) by the symmetric weighted interior penalty DG method, see for example [10, 13]:

Find $u_h^* \in {{V}_{h}}$ such that

4 $$\begin{aligned} \mathcal {A}_h({u_h^*}, \, {v_h}) = (f,v_h)_\Omega , \quad \forall v_h \in {{V}_{h}}, \end{aligned}$$

where

$$\begin{aligned} \begin{array}{ll} \mathcal {A}_h({u}, \, {v}) &{}\equiv \sum \limits _{{K}\in {\mathcal {T}_{h}}}\left( \rho \, \nabla u,\nabla v\right) _{K} + \sum \limits _{e\in \mathcal {E}_{h}}\langle \gamma [{u}],[{v}]\rangle _{e}\\ &{} \quad - \sum \limits _{e\in \mathcal {E}_{h}}(\langle [{u}],\left\{ {\rho \nabla v}\right\} _\omega \rangle _{e} + \langle \left\{ {\rho \nabla u}\right\} _\omega ,[{v}]\rangle _{e}). \end{array} \end{aligned}$$

Here for ${K}\in {\mathcal {T}_{h}}$ and $e \in \mathcal {E}_{h}$ we use standard notation

$$\begin{aligned} \left( u,v\right) _{K} = \int _{K}u\, v \, dx \quad \text {and} \quad \langle u,v\rangle _{e} = \int _e u\, v \, d\sigma . \end{aligned}$$

In what follows we shall assume that $\rho$ is piecewise constant on ${\mathcal {T}_{h}}$ (equal to its average value on each element), thus $\rho _i := \rho _{|_{{K}_i}}$ is constant for $i=1,\ldots , N_h$. On $e = \partial {K}_i \cap \partial {K}_j$ we set

$$\begin{aligned} \gamma = \dfrac{\delta }{h_e}\dfrac{\rho _i \rho _j}{\rho _i+\rho _j}, \quad \left\{ {\rho \nabla u}\right\} _\omega = \omega _j \rho _i \nabla u_i + \omega _i \rho _j \nabla u_j, \quad [{u}] = u_i\,n_i + u_j\, n_j, \end{aligned}$$

with $\omega _j = \rho _j/(\rho _i+\rho _j)$. The unit normal vector pointing outward ${K}_i$ is denoted by $n_i$. On $e$ which lies on the boundary of $\Omega$ and belongs to a face of ${K}_i$, we set $\left\{ {\rho \nabla u}\right\} _\omega = \rho _i\nabla u_i$, $[{u}] = u_i\,n_i$ and $\gamma = \delta \rho _i / h_e$.

For sufficiently large $\delta$ the discrete problem (4) is well-defined, according to the following lemma:

#### **Lemma 1**

([10, 13]) There exists positive $\delta _0$ such that if $\delta \ge \delta _0$ the bilinear form $\mathcal {A}_h({\cdot }, \, {\cdot })$ is symmetric, positive definite, and spectrally equivalent to a simplified form

$$\begin{aligned} A_h({u}, \, {v}) = \sum _{{K}\in {\mathcal {T}_{h}}}\left( \rho \, \nabla u,\nabla v\right) _{K} + \sum _{e\in \mathcal {E}_{h}}\langle \gamma [{u}],[{v}]\rangle _{e}, \end{aligned}$$

that is, there holds

5 $$\begin{aligned} \mathcal {A}_h({u}, \, {u}) \simeq A_h({u}, \, {u}) \quad \forall u \in V_h. \end{aligned}$$

In what follows we shall take as granted that $\delta$ is a fixed parameter such that $\delta \ge \delta _0$. The seminorm of a function $f$ from the Sobolev space $H^s(U)$ will be denoted by $|{f}|_{{H^s(U)}}$. For short, the $L^2$-norm of $f$ will then be denoted by $|{f}|_{{0,U}}$ or simply $|f|_U$. We also define the broken norm $||u||_{1,h}$ by the identity $||u||_{1,h}^2 = A_h({u}, \, {u})$. Then there holds the following approximation result:

#### **Lemma 2**

([10, 13]) Let $u^*\in H^1_0(\Omega )$ be the solution of (1), and let $u_h^*\in {{V}_{h}}$ satisfy the discrete problem (4). If $u^*_{|_{{K}}} \in H^2({K})$ for all ${K}\in {\mathcal {T}_{h}}$, then

$$\begin{aligned} ||u^*-u_h^*||_{1,h}^2 \lesssim \sum _{{K}\in {\mathcal {T}_{h}}}h_{{K}}^2\, \rho _{|_{K}}\, |u^*|_{H^2({K})}^2. \end{aligned}$$

## Additive Schwarz method with small subdomains

The condition number of the discrete problem (4) can be prohibitively large, affected by both the fine mesh size and by the magnitude of jumps in $\rho$. Thus, for an iterative solution of (4), some preconditioning is necessary. In this section we consider an ASM whose ingredients are: a large number of local solvers on relatively small nonoverlapping subdomains, and a coarse grid solver defined on a possibly much coarser grid.

Let us therefore introduce $\mathcal {T}_{H}$ as a partition of $\Omega$ into $N_H$ disjoint open polyhedral subdomains $\Omega _i$, $i=1,\ldots ,N_H$, such that $\bar{\Omega } = \bigcup _{i=1,\ldots ,N_H}\bar{\Omega }_i$ and that each $\Omega _i$ is a union of certain elements from the fine mesh ${\mathcal {T}_{h}}$, which we will denote ${\mathcal {T}_{h}}(\Omega _i) = \{{K}\in {\mathcal {T}_{h}}: {K}\subset \Omega _i\}$. We set $H_i = {\text {diam}}(\Omega _i)$ and $H= (H_1,\ldots , H_{N_H})$. We shall refer to this partition as the “local solvers’ grid”, because our ASM is going to solve (in parallel) local problems defined on the fine grid restricted to each of these subdomains.

Next, let $\mathcal {T}_\mathcal {H}$ be a division of $\Omega$ into $N_\mathcal {H}$ disjoint open polyhedral regions ${D}_n$, $n=1,\ldots ,N_\mathcal {H}$, such that $\bar{\Omega } = \bigcup _{n=1,\ldots ,N_\mathcal {H}}\bar{{D}}_n$ and that each ${D}_n$ is a union of certain elements from the local solvers’ grid $\mathcal {T}_{H}$. We set $\mathcal {H}_n = {\text {diam}}({D}_n)$ and then $\mathcal {H}=(\mathcal {H}_1,\ldots , \mathcal {H}_{N_\mathcal {H}})$. We shall call this partition the “coarse grid”.

We clearly have $N_\mathcal {H}\le N_H\le N_h$ and

$$\begin{aligned} \mathcal {T}_\mathcal {H}\subseteq \mathcal {T}_{H}\subseteq {\mathcal {T}_{h}}\end{aligned}$$

(inclusions understood in the sense of subsequent refinements of the coarsest partitioning), and $\max h \le \max H\le \max \mathcal {H}.$ Note that in [1, 14] the inclusions come in a different order: $\mathcal {T}_{H}\subseteq \mathcal {T}_\mathcal {H}\subseteq {\mathcal {T}_{h}}$, making the use of very small subdomains infeasible due to excessive increase of the coarse space dimension.

**Fig. 1:**
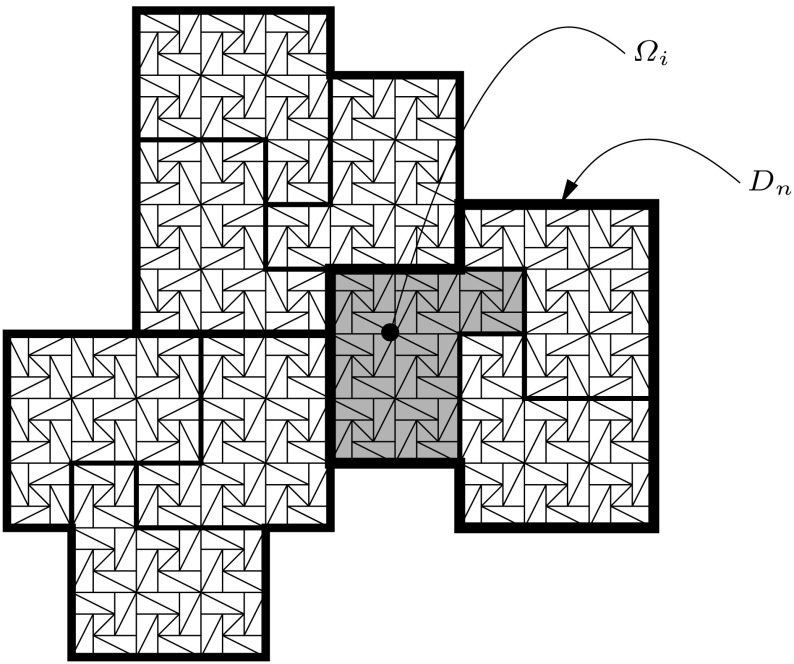
Relationship between ${\mathcal {T}_{h}}$, $\mathcal {T}_{H}$, $\mathcal {T}_\mathcal {H}$. *Thin lines* correspond to the fine grid ${\mathcal {T}_{h}}$. Local solvers’ grid $\mathcal {T}_{H}$ is marked with lines of medium width; an example local solvers’ subdomain $\Omega _i$ is *grayed*. Coarse partition $\mathcal {T}_\mathcal {H}$—here consisting of three local solvers’ subdomains per its element—is marked with *thickest lines*

An example partitioning of $\Omega$ into fine, coarse and local solvers’ grids is shown in Fig. 1.

For $S$ belonging either to $\mathcal {T}_{H}$ or to $\mathcal {T}_\mathcal {H}$ let us introduce the notation referring to the skeleton of the fine mesh restricted only to $S$:

$$\begin{aligned} \mathcal {E}_{h}(S) = \{e \in \mathcal {E}_{h}: e \subset \bar{S} \} \end{aligned}$$

and its boundary and interior parts as well:

$$\begin{aligned} \mathcal {E}_{h}^\partial (S) = \{e \in \mathcal {E}_{h}(S) : e \subset \partial {S} \}, \quad {\mathcal {E}_{h}^{0}}(S) = \mathcal {E}_{h}(S){\setminus } \mathcal {E}_{h}^\partial (S). \end{aligned}$$

Finally, let us collect all fine grid faces belonging to the boundaries of elements of $\mathcal {T}_{H}$ and $\mathcal {T}_\mathcal {H}$, respectively, to denote the skeletons of $\mathcal {T}_{H}$ and $\mathcal {T}_\mathcal {H}$:

$$\begin{aligned} \mathcal {E}_{H}= \bigcup _{i=1}^{N_H} \mathcal {E}_{h}^\partial (\Omega _i) \quad \text {and} \quad \mathcal {E}_{\mathcal {H}}= \bigcup _{n=1}^{N_\mathcal {H}} \mathcal {E}_{h}^\partial ({D}_n). \end{aligned}$$

We start the definition of the ASM by introducing a decomposition of $V_h$:

6 $$\begin{aligned} V_h = V_0 + \sum _{i=1}^{N_H} V_i \end{aligned}$$

where the coarse space is

$$\begin{aligned} V_0 = \{v\in V_h : v_{|_{{D}_n}} \in {\mathcal {P}_{0}} \text { for all } n = 1, \ldots , N_\mathcal {H}\} \end{aligned}$$

and for $i=1,\ldots ,N_H$ the local spaces are

7 $$\begin{aligned} V_i = \{v\in V_h : v_{|_{\Omega _j}} = 0 \text { for all } j \ne i\}. \end{aligned}$$

Note that ${{V}_{h}}$ is a direct sum of the local spaces. By choosing the lowest order coarse space, we reduce its dimension and, in consequence, the cost of solving the coarse problem.

Since now on, for $\varphi$ defined on $\Omega$, if necessary we shall write $\varphi _i$ to denote the restriction of $\varphi$ to $\Omega _i$:

$$\begin{aligned} \varphi _i := \varphi _{|_{\Omega _i}}. \end{aligned}$$

Observe that this brings a change from the notation of Sect. 2.1.

Using decomposition (6) we define local operators $T_i : V_h \rightarrow V_i$, $i = 1,\ldots , N_H$, by “inexact” solvers

$$\begin{aligned} A_h({T_iu}, \, {v}) = \mathcal {A}_h({u}, \, {v}) \quad \forall v \in V_i, \end{aligned}$$

so that on each subdomain one has to solve only a relatively small system of linear equations for $u_i = T_iu{|_{\Omega _i}}$ such that for all $v_i \in V_i$

$$\begin{aligned} \sum _{{K}\in {\mathcal {T}_{h}}(\Omega _i)} \left( \rho \, \nabla u_i,\nabla v_i\right) _{K} + \sum _{e \in {\mathcal {E}_{h}^{0}}(\Omega _i)} \langle \gamma [{u_i}],[{v_i}]\rangle _{e} + \sum _{e \in \mathcal {E}_{h}^\partial (\Omega _i)} \int _e {\gamma u_i}{v_i} = \mathcal {A}_h({u}, \, {v_i}). \end{aligned}$$

(For $j\ne i$ we set $(T_iu)_{|_{\Omega _j}} = 0$.) The coarse solve operator is $T_0 : V_h \rightarrow V_0$ defined analogously as

$$\begin{aligned} A_h({T_0u}, \, {v_0}) = \mathcal {A}_h({u}, \, {v_0}) \quad \forall v_0 \in V_0. \end{aligned}$$

Note that on $V_0$, the approximate form $A_h({\cdot }, \, {\cdot })$ coincides with $\mathcal {A}_h({\cdot }, \, {\cdot })$ and simplifies to

$$\begin{aligned} A_h({u_0}, \, {v_0}) = \sum _{e\in \mathcal {E}_{\mathcal {H}}}\langle \gamma [{u_0}],[{v_0}]\rangle _{e} \quad \forall u_0,v_0\in V_0. \end{aligned}$$

Finally, the preconditioned operator is

8 $$\begin{aligned} T = T_0 + \sum _{i=1}^{N_H} T_i. \end{aligned}$$

In order to formulate the condition number estimate result for $T$, we make two additional assumptions, that the elements of partition $\mathcal {T}_\mathcal {H}$ do not differ too much in shape, as well as those in $\mathcal {T}_{H}$:

1. There exists a reference simply-connected polyhedron $\hat{{D}}\subset R^d$ to which all coarse grid partition elements ${D}_n\in \mathcal {T}_\mathcal {H}$ are affinely homeomorphic and the aspect ratios of ${D}_n$, $n=1,\ldots ,N_\mathcal {H}$, are uniformly bounded with constant independent of $\mathcal {H}$, $H$ and $h$. (An example of $\mathcal {T}_\mathcal {H}$ which satisfies this condition is a partition which forms a simplicial, shape-regular, not necessarily matching, partition of $\Omega$.)
2. There exists a reference simply-connected, polyhedral structure $\hat{\Omega }\subset R^d$ to which all local solvers’ grid subdomains $\Omega _i \in \mathcal {T}_{H}$ are affinely homeomorphic and the aspect ratios of $\Omega _i$, $i=1,\ldots ,N_H$, are uniformly bounded with constant independent of $\mathcal {H}$, $H$ and $h$. Moreover, the number of neighboring regions in $\mathcal {T}_{H}$ is uniformly bounded by an absolute constant $\mathcal {N}$. (An example of $\mathcal {T}_{H}$ which satisfies this condition is a partition which forms a simplicial, shape-regular, not necessarily matching, locally uniform partition of $\Omega$.)

Assumptions (A1) and (A2) are required for Poincaré’s and trace inequalities in the proof of Theorem 1.

### **Theorem 1**

In addition to our general assumptions of Sect. 2, suppose that both (A1) and (A2) hold. Then $T$ defined in (8) is symmetric with respect to $\mathcal {A}_h({\cdot }, \, {\cdot })$ and

$$\begin{aligned} \beta ^{-1} \mathcal {A}_h({u}, \, {u}) \lesssim \mathcal {A}_h({Tu}, \, {u}) \lesssim \mathcal {A}_h({u}, \, {u}) \quad \forall u \in {{V}_{h}}, \end{aligned}$$

where

$$\begin{aligned} \beta = \max _{n=1,\ldots ,N_\mathcal {H}} \frac{\mathcal {H}_n^2}{\underline{h H}_n} \, \dfrac{{R}_n}{\underline{{R}}_n} + \max _{i=1,\ldots ,N_H} \dfrac{H_i}{\underline{h}_i} \dfrac{{r}_i}{\underline{{r}}_i} \end{aligned}$$

with mesh-dependent parameters defined as

$$\begin{aligned} \underline{h}_i&= \min \{h_{K}: {K}\in {\mathcal {T}_{h}}(\Omega _i)\}, \\ \underline{h H}_n&= \min \{\underline{h}_iH_i : \Omega _i \subset {D}_n, \, i = 1,\ldots N_H\}, \end{aligned}$$

and coefficient-dependent parameters defined as

$$\begin{aligned} \underline{{r}}_i&= \min \rho _{|_{\Omega _i}},&{r}_i&= \max \{ \rho _{|_e} : e\in \mathcal {E}_{h}^\partial (\Omega _i)\},\\ \underline{{R}}_n&= \min \rho _{|_{{D}_n}},&{R}_n&= \max \{{r}_i : \Omega _i \subset {D}_n, i = 1,\ldots N_H\}.\\ \end{aligned}$$

Therefore, the condition number of the preconditioned operator $T$ is $O(\beta )$.

Before proceeding with the proof of Theorem 1, we state a useful trace inequality result for functions in ${{V}_{h}}$ (see also [14], where the estimate of Lemma 3 is proved for a star-like region $\Omega _i$; here we do not need this assumption):

### **Lemma 3**

Under the assumptions of Sect. 2 together with (A2), for any $i=1,\ldots N_H$ there holds

$$\begin{aligned} |{v}|_{{0,\partial \Omega _i}}^2 \lesssim \frac{1}{H_i} |{v}|_{{0,\Omega _i}}^2 + H_i \left( \sum _{{K}\in {\mathcal {T}_{h}}(\Omega _i)} |{\nabla v}|_{0,{K}}^2 + \sum _{e\in {\mathcal {E}_{h}^{0}}(\Omega _i)} \dfrac{1}{h_e}|{[{v}]}|_{0,e}^2\right) \quad \forall v\in {{V}_{h}}. \end{aligned}$$

### *Proof*

Let us fix $i=1,\ldots N_H$ and let us denote by $\mathcal {T}_{\hat{h}}$ the fine mesh ${\mathcal {T}_{h}}(\Omega _i)$ affinely mapped onto $\hat{\Omega }$. It suffices to prove that for $v\in V_{\tilde{h}}$,

9 $$\begin{aligned} |{v}|_{{0,\partial \hat{\Omega }}}^2 \lesssim |{v}|_{{0,\hat{\Omega }}}^2 + ||v||_{1,\hat{h},\hat{\Omega }}^2 \end{aligned}$$

on the reference domain $\hat{\Omega }$, where for short we denote

$$\begin{aligned} ||v||_{1,\hat{h},\hat{\Omega }}^2 = \sum _{{K}\in \mathcal {T}_{\hat{h}}} |{\nabla v}|_{0,{K}}^2+ \sum _{e\in {\mathcal {E}_{\hat{h}}^0}(\hat{\Omega })} \dfrac{1}{\hat{h}_e}|{[{v}]}|_{0,e}^2, \end{aligned}$$

and then apply the standard scaling argument. By (A2) and the assumptions on ${\mathcal {T}_{h}}$ of Sect. 2.1, $\mathcal {T}_{\hat{h}}$ is also shape- and contact-regular, with relevant constants independent of $h,H,\mathcal {H}$. Thus, $\mathcal {T}_{\hat{h}}$ admits a shape-regular matching submesh $\mathcal {T}_{\tilde{h}}$, so it is sufficient to prove (9) on $\mathcal {T}_{\tilde{h}}$.

Our proof will use technical tools and results from [4]. Let us consider two subspaces of $V_{\tilde{h}}$: the space $V_{\tilde{h}}^1$ of nonconforming $P_1$ finite elements [7] on $\mathcal {T}_{\tilde{h}}$, and $\tilde{W}_{\tilde{h}}$—the continuous Lagrange finite element space consisting of piecewise polynomials from $P_2$ (in 2-D) or $P_3$ (in 3-D).

Let ${\mathcal {I}}:V_{\tilde{h}}\rightarrow V_{\tilde{h}}^1$ and ${E}: V_{\tilde{h}}^1\rightarrow \tilde{W}_{\tilde{h}}$ be operators defined in [4, Eq. (2.1) and (3.1)]. Let us recall that ${\mathcal {I}}v$ is defined by its values at the centers of the faces

$$\begin{aligned} ({\mathcal {I}}v )(c) = \dfrac{1}{|e|}\int _e \left\{ {v}\right\} \, ds \end{aligned}$$

where $c$ denotes the center of face $e$. The enriching operator ${E}$ is defined by the nodal values of $v$, so that in node $p$,

$$\begin{aligned} ({E}v)(p) = \dfrac{1}{n_p} \sum _{{K}\in \mathcal {V}(p)} v_{|_{K}}(p), \end{aligned}$$

where $\mathcal {V}(p)$ is the set of elements in $\mathcal {T}_{\tilde{h}}$ sharing node $p$, and $n_p$ is the number of these elements.

We will use the following estimates from [4, Eq. (2.8), (2.9), (3.10)–(3.11) and estimates from Example 4.2]:

10 $$\begin{aligned} \sum _{{K}\in \mathcal {T}_{\tilde{h}}}|{\nabla {\mathcal {I}}v}|_{0,{K}}^2 \lesssim ||v||_{1,\tilde{h},\hat{\Omega }}^2, \quad \sum _{{K}\in \mathcal {T}_{\tilde{h}}}\tilde{h}_{K}^{-1}|{v- {\mathcal {I}}v}|_{0,{K}}^2 \lesssim ||v||_{1,\tilde{h},\hat{\Omega }}^2 \end{aligned}$$

for all $v\in V_{\tilde{h}}^1$, and

11 $$\begin{aligned} |{{E}v}|_{{\hat{\Omega }}}^2 \lesssim |{v}|_{{\hat{\Omega }}}^2, \quad |{\nabla {E}v}|_{{\hat{\Omega }}}^2 \lesssim \sum _{{K}\in \mathcal {T}_{\tilde{h}}} |{\nabla v}|_{0,{K}}^2, \quad |{{E}v - v}|_{{\partial \hat{\Omega }}}^2 \lesssim \sum _{{K}\in \mathcal {T}_{\tilde{h}}} \tilde{h}_{K}|{\nabla v}|_{0,{K}}^2 \end{aligned}$$

for all $$v\in \tilde{W}_{\tilde{h}}$$.

For $v\in V_{\tilde{h}}$ we have

12 $$\begin{aligned} |{v}|_{{0,\partial \hat{\Omega }}}^2 \lesssim |{v-{\mathcal {I}}v}|_{{0,\partial \hat{\Omega }}}^2 + |{{\mathcal {I}}v - {E}({\mathcal {I}}v)}|_{{0,\partial \hat{\Omega }}}^2 + |{{E}({\mathcal {I}}v)}|_{{0,\partial \hat{\Omega }}}^2. \end{aligned}$$

By trace inequality and the last inequality of (10),

$$\begin{aligned} |{v-{\mathcal {I}}v}|_{{0,\partial \hat{\Omega }}}^2 \lesssim \sum _{{K}\in \mathcal {T}_{\tilde{h}}} \tilde{h}_{K}^{-1}|{v - {\mathcal {I}}v}|_{0,{K}}^2 \lesssim ||v||_{1,\tilde{h},\hat{\Omega }}^2. \end{aligned}$$

Using the last estimate of (11) and then the first from (10) we get

$$\begin{aligned} |{{\mathcal {I}}v - {E}({\mathcal {I}}v)}|_{{0,\partial \hat{\Omega }}}^2 \lesssim \sum _{{K}\in \mathcal {T}_{\tilde{h}}}|{\nabla {\mathcal {I}}v}|_{0,{K}}^2 \lesssim ||v||_{1,\tilde{h},\hat{\Omega }}^2. \end{aligned}$$

Finally, as ${E}({\mathcal {I}}v)\in H^1(\hat{\Omega })$, we can apply standard trace inequality and use (11) and (10), to conclude that

$$\begin{aligned} |{{E}({\mathcal {I}}v)}|_{{0,\partial \hat{\Omega }}}^2 \lesssim |{ {E}({\mathcal {I}}v)}|_{{0,\hat{\Omega }}}^2 + |{\nabla {E}({\mathcal {I}}v)}|_{{0,\hat{\Omega }}}^2 \lesssim |{ v}|_{{0,\hat{\Omega }}}^2 + ||v||_{1,\tilde{h},\hat{\Omega }}^2. \end{aligned}$$

Substituting the above inequalities into (12) we arrive at (9). $\square$

### *Proof (of Theorem 1)*

We follow the abstract theory of additive Schwarz methods, cf. [16, Theorem 2.7], and prove three key properties for $T_0,T_1,\ldots ,T_{N_H}$:

*Strengthened Cauchy–Schwarz inequality* It is straightforward to verify that if $u\in V_i$ and $v \in V_j$ with $i,j \in \{1,\ldots ,N_H\}$ then $\mathcal {A}_h({u}, \, {v}) = 0$, if only $\Omega _i$ and $\Omega _j$ are not neighbors, i.e. they do not share a face in $\mathcal {E}_{H}^0$. Thus, we conclude that then

$$\begin{aligned} \mathcal {A}_h({u}, \, {v}) \le \epsilon _{ij}\mathcal {A}_h({u}, \, {u})^{1/2} \mathcal {A}_h({v}, \, {v}) ^{1/2} \end{aligned}$$

and the spectral radius of $\epsilon = (\epsilon _{ij})_{i,j=1}^{N_H}$ is bounded by $\mathcal {N}$—the maximum number of neighbors of any element in $\mathcal {T}_{H}$. According to assumption (A2) $\mathcal {N}$ is bounded independently of the mesh parameters $h$ and $H$ (and, clearly, independently of other parameters of the problem).

*Local stability* For all $i = 0,1,\ldots ,N_H$

$$\begin{aligned} \mathcal {A}_h({u}, \, {u}) \le \omega \, A_h({u}, \, {u}) \quad \forall u \in V_i, \end{aligned}$$

with absolute constant $\omega$ independent of $\rho$, $h$, $H$ and $\mathcal {H}$. This is an obvious consequence of (5); in addition, $\omega \ge 1$, because on $V_0$ both forms coincide.

*Stable decomposition* We have to prove that there exist $C_0$ independent of $h,H,\mathcal {H},\rho$, and a decomposition of $u\in {{V}_{h}}$,

13 $$\begin{aligned} u = \sum _{i=0}^{N_H} u^{(i)} \quad \text {with } u^{(i)} \in V_i, \end{aligned}$$

such that

14 $$\begin{aligned} \sum _{i=0}^{N_H}A_h({u^{(i)}}, \, {u^{(i)}}) \le C_0^2 \mathcal {A}_h({u}, \, {u}) \quad \forall u \in {{V}_{h}}. \end{aligned}$$

In order to construct the stable decomposition of $u\in {{V}_{h}}$, we first define $u^{(0)}\in V_0$ such that on each ${D}_n\in \mathcal {T}_\mathcal {H}$, $n = 1,\ldots ,N_\mathcal {H}$,

$$\begin{aligned} u^{(0)}_{|_{{D}_n}} = \bar{u}_{(n)} = \dfrac{1}{|{D}_n|} \int _{{D}_n} u\, dx. \end{aligned}$$

Note that $u^{(0)}$ is constant on the local solvers’ subdomains $\Omega _i$, $i = 1,\ldots ,N_H$, as well.

Next, for $i = 1,\ldots ,N_H$, we define $u^{(i)}\in V_i$, as a zero-extension of the restriction of $u-u^{(0)}$ to $\Omega _i$:

$$\begin{aligned} u^{(i)} = {\left\{ \begin{array}{ll} u - u^{(0)} \quad &{}\text {on } \Omega _i,\\ 0 \quad &{}\text {elsewhere}. \end{array}\right. } \end{aligned}$$

We obviously have, since $u^{(0)}$ is subdomainwise constant and its jumps cannot occur inside any element of $\mathcal {T}_{H}$,

$$\begin{aligned} \sum _{i=1}^{N_H} A_h({u^{(i)}}, \, {u^{(i)}})&= \sum _{i=1}^{N_H} \sum _{{K}\in {\mathcal {T}_{h}}(\Omega _i)} \left( \rho \nabla u,\nabla u\right) _{K} + \sum _{i=1}^{N_H} \sum _{e\in {\mathcal {E}_{h}^{0}}(\Omega _i)} \gamma |{[{u}]}|_{0,e}^2 \\&\quad + \sum _{i=1}^{N_H} \sum _{e\in \mathcal {E}_{h}^\partial (\Omega _i)} \gamma |{(u-u^{(0)})_i}|_{0,e}^2 \\&\le A_h({u}, \, {u}) + \sum _{i=1}^{N_H} \sum _{e\in \mathcal {E}_{h}^\partial (\Omega _i)} \gamma |{(u-u^{(0)})_i}|_{0,e}^2. \end{aligned}$$

Let us consider subdomain $\Omega _i$. Then, it follows that on $e \in \mathcal {E}_{h}^\partial (\Omega _i)$ such that $e = \partial {K}_I\cap \partial {K}_J$ and ${K}_I \in {\mathcal {T}_{h}}(\Omega _i)$, ${K}_J \in {\mathcal {T}_{h}}(\Omega _j)$ there holds

$$\begin{aligned} \gamma = \dfrac{\delta }{h_e} \dfrac{\rho _{|_{{K}_I}} \rho _{|_{{K}_J}}}{\rho _{|_{{K}_I}}+\rho _{|_{{K}_J}}} \lesssim \dfrac{\delta }{h_{{K}_I}} \rho _{|_{{K}_I}} \lesssim \dfrac{{r}_i}{\underline{h}_{i}} \end{aligned}$$

and analogously, if $e$ lies on the boundary of $\Omega$, then

$$\begin{aligned} \gamma = \dfrac{\delta \rho _{|_{{K}_I}} }{h_e} \lesssim \dfrac{{r}_i}{\underline{h}_{i}}, \end{aligned}$$

so that

$$\begin{aligned} \sum _{e\in \mathcal {E}_{h}^\partial (\Omega _i)} \gamma |{(u-u^{(0)})_i}|_{0,e}^2 \lesssim \dfrac{{r}_i}{\underline{h}_i} \sum _{e\in \mathcal {E}_{h}^\partial (\Omega _i)} |{(u-u^{(0)})_i}|_{0,e}^2 = \dfrac{{r}_i}{\underline{h}_i} |{(u-u^{(0)})_i}|_{0,{\partial \Omega _i}}^2. \end{aligned}$$

From (A2) it follows that we can apply the trace inequality of Lemma 3 to $u-u^{(0)}_i$ and obtain

$$\begin{aligned}&|{(u-u^{(0)})_i}|_{0,{\partial \Omega _i}}^2\\&\quad \lesssim \dfrac{1}{H_i}|{u-u^{(0)}}|_{{\Omega _i}}^2 + H_i \left( \sum _{{K}\in {\mathcal {T}_{h}}(\Omega _i)} |{\nabla u}|_{0,{K}}^2. + \sum _{e\in {\mathcal {E}_{h}^{0}}(\Omega _i)} \dfrac{1}{h_e} |{[{u}]}|_{0,e}^2 \right) , \end{aligned}$$

because again $[{u^{(0)}_i}]$ vanishes on the inner skeleton ${\mathcal {E}_{h}^{0}}(\Omega _i)$. Then,

$$\begin{aligned} \sum _{i=1}^{N_H} \sum _{e\in \mathcal {E}_{h}^\partial (\Omega _i)} \gamma |{(u-u^{(0)})_i}|_{0,e}^2 \lesssim \sum _{i=1}^{N_H} \dfrac{{r}_i}{\underline{h}_i H_i} |{u-u^{(0)}}|_{{\Omega _i}}^2 + \max _{i=1,\ldots ,N_H} \dfrac{{r}_i}{\underline{{r}}_i}\dfrac{H_i}{\underline{h}_i} A_h({u}, \, {u}). \end{aligned}$$

Now,

$$\begin{aligned} \sum _{i=1}^{N_H} \dfrac{{r}_i}{\underline{h}_i H_i} |{u-u^{(0)}}|_{{\Omega _i}}^2&= \sum _{n=1}^{N_\mathcal {H}} \sum _{i: \Omega _i \subset {D}_n} \dfrac{{r}_i}{\underline{h}_i H_i} |{u-u^{(0)}}|_{{\Omega _i}}^2 \\&\le \sum _{n=1}^{N_\mathcal {H}}\dfrac{{R}_n}{\underline{h H}_n} |{u-\bar{u}_{(n)}}|_{{{D}_n}}^2. \end{aligned}$$

Applying Poincaré’s inequality for discontinuous finite element functions [4, Theorem 5.1] (see also [8, 14]) on the matching submesh of ${D}_n$ and then using the shape- and contact-regularity assumption followed by the scaling argument we get

$$\begin{aligned} |{u-\bar{u}_{(n)}}|_{{{D}_n}}^2&\lesssim \mathcal {H}_n^2 \left( \sum _{{K}\in {\mathcal {T}_{h}}({D}_n)}|{\nabla u}|_{0,{K}}^2 + \sum _{e \in {\mathcal {E}_{h}^{0}}({D}_n)} \dfrac{1}{h_e} |{[{u}]}|_{0,e}^2\right) \\&\lesssim \mathcal {H}_n^2 \left( \sum _{i: \Omega _i \subset {D}_n} \sum _{{K}\in {\mathcal {T}_{h}}(\Omega _i)}|{\nabla u}|_{0,{K}}^2 + \sum _{e \in {\mathcal {E}_{h}^{0}}({D}_n)} \dfrac{1}{h_e} |{[{u}]}|_{0,e}^2 \right) . \end{aligned}$$

Because on $e \in {\mathcal {E}_{h}^{0}}(D_n)$ such that $e = \partial {K}_I \cap \partial {K}_J$ there holds $\min \{\rho _{{K}_I},\rho _{{K}_J}\} \le 2 \rho _{{K}_I}\rho _{{K}_J}/(\rho _{{K}_I} + \rho _{{K}_J})$, we conclude that

$$\begin{aligned} \underline{{R}}_n \le \frac{2 \gamma h_e}{\delta } \quad \text { on } {\mathcal {E}_{h}^{0}}(D_n), \end{aligned}$$

and therefore

$$\begin{aligned} |{u-\bar{u}_{(n)}}|_{{{D}_n}}^2&\lesssim \mathcal {H}_n^2 \left( \sum _{i: \Omega _i \subset {D}_n} \dfrac{1}{\underline{{r}}_i} \sum _{{K}\in {\mathcal {T}_{h}}(\Omega _i)}\rho _{|_{K}}|{\nabla u}|_{0,{K}}^2 + \dfrac{1}{\underline{{R}}_n}\sum _{e \in {\mathcal {E}_{h}^{0}}({D}_n)} \gamma |{[{u}]}|_{0,e}^2\right) \\&\lesssim \mathcal {H}_n^2 \, \dfrac{1}{\underline{{R}}_n} \sum _{i: \Omega _i \subset {D}_n}\left( \sum _{{K}\in {\mathcal {T}_{h}}(\Omega _i)}\rho _{|_{K}}|{\nabla u}|_{0,{K}}^2 +\sum _{e \in \mathcal {E}_{h}(\Omega _i)} \gamma |{[{u}]}|_{0,e}^2\right) . \end{aligned}$$

From the above we can estimate

$$\begin{aligned} \sum _{i=1}^{N_H} \sum _{e\in \mathcal {E}_{h}^\partial (\Omega _i)} \gamma |{(u-u^{(0)})_i}|_{0,e}^2 \lesssim \left( \max _{n=1,\ldots ,N_\mathcal {H}} \frac{\mathcal {H}_n^2}{\underline{h H}_n} \, \dfrac{{R}_n }{\underline{{R}}_n} + \max _{i=1,\ldots ,N_H} \dfrac{H_i}{\underline{h}_i} \dfrac{{r}_i}{\underline{{r}}_i} \right) A_h({u}, \, {u}) \end{aligned}$$

and in consequence

$$\begin{aligned} \sum _{i=1}^{N_H} A_h({u^{(i)}}, \, {u^{(i)}}) \lesssim \beta A_h({u}, \, {u}) \lesssim \beta \mathcal {A}_h({u}, \, {u}), \end{aligned}$$

the latter inequality following from Lemma 1.

It remains to bound $A_h({u^{(0)}}, \, {u^{(0)}})$. We have

$$\begin{aligned} A_h({u^{(0)}}, \, {u^{(0)}}) = \sum _{e\in \mathcal {E}_{\mathcal {H}}}\gamma |{[{u^{(0)}}]}|_{0,e}^2 \lesssim \sum _{e\in \mathcal {E}_{\mathcal {H}}}\gamma |{[{u-u^{(0)}}]}|_{0,e}^2 +\sum _{e\in \mathcal {E}_{\mathcal {H}}} \gamma |{[{u}]}|_{0,e}^2 \end{aligned}$$

For any $e\!\in \! \mathcal {E}_{\mathcal {H}}$ such that $e = \partial {K}_I \cap \partial {K}_J$ and ${K}_I \!\in \!{\mathcal {T}_{h}}(\Omega _i)$ and ${K}_J \!\in \!{\mathcal {T}_{h}}(\Omega _j)$ we have

$$\begin{aligned} |{[{u-u^{(0)}}]}|_{0,e}^2 \lesssim |{(u-u^{(0)})_i}|_{0,e}^2 + |{(u-u^{(0)})_j}|_{0,e}^2, \end{aligned}$$

so that

$$\begin{aligned} \sum _{e\in \mathcal {E}_{\mathcal {H}}}\gamma |{[{u^{(0)}}]}|_{0,e}^2 \lesssim \sum _{i=1}^{N_H} \sum _{e\in \mathcal {E}_{h}^\partial (\Omega _i)} \gamma |{(u-u^{(0)})_i}|_{0,e}^2+ A_h({u}, \, {u}) \end{aligned}$$

Since the first term has already been estimated by $\beta A_h({u}, \, {u})$, we conclude that also

$$\begin{aligned} A_h({u^{(0)}}, \, {u^{(0)}}) \lesssim \beta \mathcal {A}_h({u}, \, {u}), \end{aligned}$$

which completes the proof.

### *Remark 1*

In (A1) or (A2) we could have assumed the existence of a finite set of several such reference structures, the number of which is another absolute constant independent of $h$, $H$, $\mathcal {H}$ and the jumps in $\rho$.

Below we prove that the convergence speed of the method is independent of the magnitude of jumps of $\rho$, if the variation of coefficient $\rho$ is assumed moderate:

- either on the skeleton of the local solvers’ partition $\mathcal {E}_{H}$,
- or on the coarse partition $\mathcal {T}_\mathcal {H}$.

Observe that then large jumps of $\rho$ are allowed, respectively, either on islands strictly inside local subdomains or on the boundaries of the coarse partition.

### **Corollary 1**

In addition to the assumptions of Theorem 1 suppose that the variation of the coefficient $\rho$ on the skeleton $\mathcal {E}_{H}$ is uniformly bounded by an absolute constant of order $1$:

15 $$\begin{aligned} \rho _{|_e} \simeq 1 \quad \forall e\in \mathcal {E}_{H}. \end{aligned}$$

Then

$$\begin{aligned} {\text {cond}}(T) = O\left( \max _{n=1,\ldots ,N_\mathcal {H}} \frac{\mathcal {H}_n^2}{\underline{h H}_n} + \max _{i=1,\ldots ,N_H} \dfrac{H_i}{\underline{h}_i} \right) . \end{aligned}$$

### *Proof*

Since $\rho$ is normalized so that $\rho \ge 1$ it follows that $1 \le \underline{{r}}_i$ and $1 \le \underline{{R}}_n.$ Therefore $1/\underline{{R}}_n \le 1$ and, since $\mathcal {E}_{h}^\partial (\Omega _i) \subset \mathcal {E}_{H}$ for $i = 1,\ldots , N_H$,

$$\begin{aligned} \dfrac{{r}_i}{\underline{{r}}_i} \le {r}_i \lesssim 1, \end{aligned}$$

so in consequence $\max _n {R}_n \lesssim 1.$ $\square$

### **Corollary 2**

In addition to the assumptions of Theorem 1 suppose that the variation of the coefficient $\rho$ is uniformly bounded by an absolute constant within elements of the coarse partition $\mathcal {T}_\mathcal {H}$:

16 $$\begin{aligned} \rho _{|_{{D}_n}} \simeq \bar{\rho }_{(n)} := \frac{1}{|{D}_n|}\int _{{D}_n}\rho \, dx, \quad n = 1,\ldots ,N_\mathcal {H}, \end{aligned}$$

(for example, $\rho$ can be piecewise constant on $\mathcal {T}_\mathcal {H}$ with arbitrary jumps across the interface $\mathcal {E}_{\mathcal {H}}$). Then

$$\begin{aligned} {\text {cond}}(T) = O\left( \max _{n=1,\ldots ,N_\mathcal {H}} \frac{\mathcal {H}_n^2}{\underline{h H}_n} + \max _{i=1,\ldots ,N_H} \dfrac{H_i}{\underline{h}_i} \right) . \end{aligned}$$

### *Proof*

By our assumption, for any $i=1,\ldots , N_H$ and for $n$ such that $\Omega _i \subset {D}_n$ we have

$$\begin{aligned} {r}_i, \, \underline{{r}}_i \simeq \bar{\rho }_{(n)}, \end{aligned}$$

so that ${r}_i/\underline{{r}}_i \lesssim 1$, and for any $n = 1,\ldots ,N_\mathcal {H}$ there holds

$$\begin{aligned} {R}_n, \, \underline{{R}}_n, \simeq \bar{\rho }_{(n)} \end{aligned}$$

as well. $$\square $$

### *Remark 2*

If we suppose that (15) or (16) is satisfied and that all meshes ${\mathcal {T}_{h}}$, $\mathcal {T}_{H}$ and $\mathcal {T}_\mathcal {H}$ are quasi-uniform, then (with a little abuse of notation) the expression for ${\text {cond}}(T)$ simplifies to

$$\begin{aligned} {\text {cond}}(T) = O\left( \frac{\mathcal {H}^2}{h\,H} \right) , \end{aligned}$$

where $h$, $H$, $\mathcal {H}$ here mean the *maximum* diameter of elements in ${\mathcal {T}_{h}}$, $\mathcal {T}_{H}$ and $\mathcal {T}_\mathcal {H}$, respectively.

If each local solvers’ subdomain reduces to a single element of the fine mesh, i.e. $\mathcal {T}_{H}= {\mathcal {T}_{h}}$, one has to solve in parallel $N_h = O(h^d)$ local problems, which are just a very simple $3\times 3$ (in 2-D) or $4\times 4$ (in 3-D) system each, while the coarse problem remains fixed with (relatively small) size $N_\mathcal {H}= O(\mathcal {H}^d)$. The price to be paid for this massive parallelism is slower (as compared to the standard approach, cf. Corollary 4 below) convergence of the iteration:

### **Corollary 3**

In addition to our general assumptions of Sect. 2, assume that $\mathcal {T}_{H}= {\mathcal {T}_{h}}$ and (A1) holds together with (16). Then

$$\begin{aligned} {\text {cond}}(T) = O\left( \max _{n=1,\ldots ,N_\mathcal {H}} \frac{\mathcal {H}_n^2}{\min _{{K}\in {\mathcal {T}_{h}}({D}_n)}h_{{K}}^2}\right) . \end{aligned}$$

### *Proof*

Assumption (A2) is automatically satisfied since $\Omega _i$ reduces to single element in ${\mathcal {T}_{h}}$, so the estimate follows directly from Corollary 2. $\square$

Theorem 1 also recovers previously known bounds, for constant or piecewise constant coefficient, in the “standard” case when $\mathcal {T}_{H}= \mathcal {T}_\mathcal {H}$ (see [1, 10, 14]), and extends them to the case of coefficient mildly varying over $\mathcal {E}_{H}$:

### **Corollary 4**

In addition to our general assumptions of Sect. 2, assume that $\mathcal {T}_{H}= \mathcal {T}_\mathcal {H}$ and (A2) holds together with either (15) or (16). Then

$$\begin{aligned} {\text {cond}}(T) = O\left( \max _{i=1,\ldots ,N_H} \frac{H_i}{\min _{{K}\in {\mathcal {T}_{h}}(\Omega _i)}h_{{K}}}\right) . \end{aligned}$$

### *Proof*

The proof follows straightforwardly from Corollaries 1 and 2 and thus is omitted. $\square$

For completeness, let us also mention the following:

### **Corollary 5**

In addition to our general assumptions of Sect. 2, assume that ${\mathcal {T}_{h}}= \mathcal {T}_{H}= \mathcal {T}_\mathcal {H}$. Then ${\text {cond}}(T) = O(1)$.

### *Proof*

Assumption (16) is automatically fulfilled on a single element, while (A1) and (A2) are trivially satisfied because of our assumptions on the fine grid in Sect. 2. The estimate then follows again from Corollary 2. $\square$

Although the condition number in Corollary 5 is independent both from $h$ and the jumps of $\rho$, the preconditioned operator $T$ is not robust in this particular case: the number of degrees of freedom in the coarse space operator $T_0$ is unacceptably large, of order $N_h$, i.e. of the same order as the original size of the discrete problem. On the other hand, $T_0$ is defined only on the space of piecewise constant functions.

## Numerical experiments

Let us choose the unit square $[0,1]^2$ as the domain $\Omega$ and for some prescribed integer $\mathcal {M}$ divide it into $N_\mathcal {H}= 2^\mathcal {M}\times 2^\mathcal {M}$ smaller squares ${D}_n$ ($n = 1,\ldots ,N_\mathcal {H}$) of equal size. This coarse grid $\mathcal {T}_\mathcal {H}$ is then refined into a uniform local solvers’ $2^M \times 2^M$ square grid $\mathcal {T}_{H}$, which in turn is further refined into a uniform fine triangulation ${\mathcal {T}_{h}}$ based on a square $2^m\times 2^m$ grid ($m\ge M \ge \mathcal {M}$) with each square split into two triangles of identical shape. Hence, the grid parameters are $h=2^{-m}$, $H= 2^{-M}$ and $\mathcal {H}=2^{-\mathcal {M}}$; obviously, there holds $\mathcal {T}_\mathcal {H}\subseteq \mathcal {T}_{H}\subseteq {\mathcal {T}_{h}}$. We discretize problem (1) on the fine mesh ${\mathcal {T}_{h}}$ using (4) with $\delta = 7$.

In the following tables we report the number of Preconditioned Conjugate Gradient iterations for operator $T$ which are required to reduce the initial Euclidean norm of the residual by a factor of $10^6$ and (in parentheses) the condition number of $T$ approximation computed from the extreme eigenvalues estimate based on the PCG coefficients, see e.g. [15, Sect. 6.7.3]. We always choose a random vector for the right hand side and zero as the initial guess.

### Dependence on $h$, $H$, $\mathcal {H}$

First, let us consider the convergence of the “massively parallel” method when $\mathcal {T}_{H}= {\mathcal {T}_{h}}$ against “standard” approach, when $\mathcal {T}_{H}= \mathcal {T}_\mathcal {H}$. For the diffusion coefficient we take an elementwise $P_0$ approximation of a continuous function $\rho (x) = x_1^2 + x_2^2 + 1$. As it turns out from Table 1, the condition number of the method considered in Corollary 3 indeed shows an $O(\mathcal {H}^2/h^2)$ behavior, as predicted, while methods which use local solves on subdomains of diameter at least $\mathcal {H}$ (e.g. [1, 14]) exhibit $O(\mathcal {H}/h)$ dependence, cf. Corollary 4 and Table 2.

**Table 1:** Dependence of the number of iterations and the condition number (in parentheses) on $\mathcal {H}=2^{-\mathcal {M}}$ and $h=2^{-m}$ for the method with $\mathcal {T}_{H}= {\mathcal {T}_{h}}$

Coarse ($\mathcal {M}$)	Fine ($m$)			
	4	5	6	7
4	$29$ ($22$)	$39$ ($40$)	$59$ ($1.1\times 10^{2}$)	$96$ ($3.8\times 10^{2}$)
5		$30$ ($23$)	$39$ ($40$)	$59$ ($1.1\times 10^{2}$)
6			$30$ ($23$)	$38$ ($40$)
7				$30$ ($23$)

Slowly varying coefficient $\rho$

**Table 2:** Dependence of the number of iterations and the condition number (in parentheses) on $\mathcal {H}=2^{-\mathcal {M}}$ and $h=2^{-m}$ for the method with $\mathcal {T}_{H}= \mathcal {T}_\mathcal {H}$

Coarse ($\mathcal {M}$)	Fine ($m$)			
	4	5	6	7
4	$27$ ($20$)	$35$ ($34$)	$46$ ($67$)	$62$ ($1.3\times 10^{2}$)
5		$28$ ($20$)	$35$ ($34$)	$46$ ($67$)
6			$28$ ($20$)	$35$ ($34$)
7				$28$ ($20$)

Slowly varying coefficient $\rho$

### Strongly discontinuous coefficient piecewise constant on $\mathcal {T}_\mathcal {H}$

Here we deal with large discontinuities in $\rho$ under restrictions of Corollary 3. The case of discontinuous coefficient when $\mathcal {T}_{H}= \mathcal {T}_\mathcal {H}$ (cf. Corollary  4) has already been treated elsewhere [12], so we restrict ourselves only to the case when $\mathcal {T}_{H}= {\mathcal {T}_{h}}$. Let us then consider $\rho$ with discontinuities aligned with an auxiliary partitioning of $\Omega$ into $4\times 4$ squares. Precisely, we introduce a red–black checkerboard coloring of this partitioning and set $\rho$ equal to $\rho _R=1$ in red regions, and the value of $\rho _{B}$ reported in Table 3 in black ones. In this way, our fine and coarse triangulations, with $m=7$ and $\mathcal {M}=4$, are always aligned with the discontinuities, as required in Corollary 3. Table 3 confirms the condition number is independent from $\rho _{B}$ in this case.

**Table 3:** Dependence of the number of iterations and the condition number (in parentheses) on the discontinuity when the coefficient is constant inside coarse partition and $\mathcal {T}_{H}={\mathcal {T}_{h}}$

$\rho _{B}$	$10^{0}$	$10^{2}$	$10^{4}$	$10^{6}$
Iter (cond)	$134$ $(3.8\times 10^{2})$	$143$ $(3.7\times 10^{2})$	$160$ $(3.7\times 10^{2})$	$181$ $(3.7\times 10^{2})$

Red–black $4\times 4$ distribution of $\rho$, aligned with the coarse grid. Fixed $\mathcal {H}/h = 8$

### Strongly discontinuous coefficient piecewise constant on ${\mathcal {T}_{h}}$

Next, we investigate elementwise discontinuous coefficient with $m=7$ and $\mathcal {M}=4$ as before, but with $\rho = 1$ on odd and $\rho = \rho _B$ on even-numbered triangles of ${\mathcal {T}_{h}}$. Table 4 shows that in this case the preconditioner fails (a dash means the method did not converge in 600 iterations) for large jumps in $\rho$. This confirms the importance of controlled variation of the coefficient inside elements over the partition of the domain.

**Table 4:** Dependence of the number of iterations and the condition number (in parentheses) on the discontinuity when the coefficient is elementwise discontinuous

$\rho _{B}$	$10^{0}$	$10^{2}$	$10^{4}$	$10^{6}$
Iter (cond)	$134$ ($3.8\times 10^{2}$)	$435$ ($3.8\times 10^{3}$)	$-$ ($3.1\times 10^{5}$)	$-$ ($2.5\times 10^{7}$)

Fixed $$\mathcal {H}/h = 8$$ and $$\mathcal {T}_{H}= {\mathcal {T}_{h}}$$

As stated in Corollary 5, in the unfeasible case when ${\mathcal {T}_{h}}= \mathcal {T}_{H}= \mathcal {T}_\mathcal {H}$, the condition number of the preconditioned system is $O(1)$ regardless of $h$ and the jumps of $\rho$ (parameters $H$ and $\mathcal {H}$ do not apply here) and this is confirmed in experiments, as reported in Table 5.

**Table 5:** Dependence of the number of iterations and the condition number (in parentheses) on the discontinuity and fine mesh size $h=2^{-m}$ when the coefficient is elementwise discontinuous and ${\mathcal {T}_{h}}= \mathcal {T}_{H}= \mathcal {T}_\mathcal {H}$

$\rho _B$	Fine ($m$)				
	3	4	5	6	7
$10^0$	40 (34)	44 (41)	45 (42)	47 (44)	47 (45)
$10^{6}$	38 (22)	41 (24)	42 (25)	66 (50)	64 (50)

### Strongly discontinuous coefficient constant on $\mathcal {E}_{H}$

**Fig. 2:**
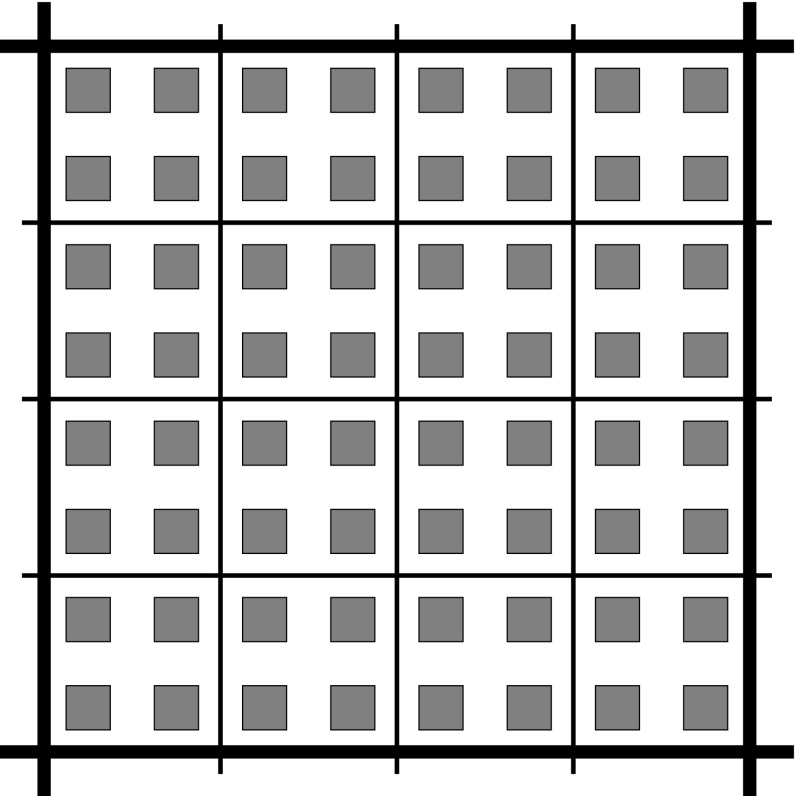
Example distribution of coefficient $\rho$, considered in Sect. 4.4, depicted inside a single square interior element of $\mathcal {T}_\mathcal {H}$ (whose edges are marked with *thick lines*) when $\mathcal {M}= 2$. In *gray islands* $\rho =10^6$; elsewhere $\rho =1$. Local solvers’ grid marked with *thinner lines* for $M=4$. Note that in presented example, $\rho$ on $\mathcal {E}_{H}$ is constant

In Corollary 1 we show that in some circumstances one can allow very large variation of $\rho$ even inside local solvers’ subdomains. Let us then choose $\rho$ constant equal to 1, except for square islands, where we set $\rho = 10^6$. The islands are centrally located in cells of a $2^5 \times 2^5$ independent division of $\Omega$ into squares. The size of every island equals half-length of the cell (see Fig. 2 for an example). In order to make the fine triangulation resolve the discontinuities of $\rho$ well, we restrict ourselves only to $m = 8$.

As it turns out from Table 6, if local solvers’ subdomains entirely cover the islands—that is, if $M \le 5$—then $\rho$ turns constant on $\mathcal {E}_{H}$ and the performance of the preconditioner is not sensitive to large jumps in the coefficients inside subdomains, which is in agreement with Corollary 1. Moreover, in the case when there are jumps across $\mathcal {E}_{H}$ and at the same time the coarse grid does not resolve the discontinuities well enough, i.e. $M > 5 > \mathcal {M}$, the condition number becomes sensitive to the coefficient jumps.

**Table 6:** Dependence of the number of iterations and the condition number (in parentheses) on $\mathcal {H}=2^{-\mathcal {M}}$ and $H=2^{-M}$ when the coefficient is discontinuous with contrast ratio $10^6$ on small islands centrally placed on a $2^5\times 2^5$ grid, cf. Fig. 2

Coarse ($\mathcal {M}$)	Local ($M$)						
	2	3	4	5	6	7	8
2	132 ($4.9\cdot 10^2$)	151 ($5.8\cdot 10^2$)	188 ($8.6\cdot 10^2$)	253 ($1.4\cdot 10^3$)	– ($5.6\cdot 10^8$)	– ($5.1\cdot 10^8$)	– ($1.4\cdot 10^9$)
3		120 ($3.1\cdot 10^2$)	129 ($3.5\cdot 10^2$)	152 ($4.7\cdot 10^2$)	– ($1.6\cdot 10^8$)	– ($1.4\cdot 10^8$)	– ($3.7\cdot 10^8$)
4			93 ($1.7\cdot 10^2$)	98 ($1.8\cdot 10^2$)	737 ($4.7\cdot 10^7$)	751 ($4.3\cdot 10^7$)	883 ($1.1\cdot 10^8$)
5				69 ($9.0\cdot 10^1$)	76 ($1.1\cdot 10^2$)	81 ($1.1\cdot 10^2$)	88 ($1.3\cdot 10^2$)
6					77 ($1.2\cdot 10^2$)	99 ($1.3\cdot 10^2$)	115 ($1.5\cdot 10^2$)
7						62 ($5.2\cdot 10^1$)	59 ($4.6\cdot 10^1$)
8							

Fine grid parameter is $h=2^{-m}$ with $m = 8$

When $\mathcal {M}\ge 5$, we are back to the case when the coefficient is constant on $\mathcal {T}_\mathcal {H}$ (i.e. the case of Corollary 2) and results from Table 6 reflect this.

## Conclusions

A nonoverlapping ASM preconditioner for symmetric interior penalty DG discretization of second order elliptic PDE with discontinuous coefficient on a nonmatching mesh ${\mathcal {T}_{h}}$ has been presented. A very large number of small local problems defined by subdomains introduced by a domain partition $\mathcal {T}_{H}$ is solved in parallel, together with one coarse problem of moderate size on a coarser partition $\mathcal {T}_\mathcal {H}$ such that $\mathcal {T}_\mathcal {H}\subseteq \mathcal {T}_{H}\subseteq {\mathcal {T}_{h}}$ in the sense of subsequent refinements. Two cases of arrangement of the coefficient discontinuity relative to partitionings $\mathcal {T}_\mathcal {H}$ or $\mathcal {T}_{H}$ have been identified, for which the condition number of the resulting system is $O(\mathcal {H}^2/hH)$ independently of the jumps of the coefficient. This property allows one to design a massively parallel method with rate of convergence independent of the jumps of the coefficient. Our result also shows that the decrease of local solvers’ subdomains size $H$ (i.e. the increase of parallelism) affects the condition number only linearly, while it turns out highly important to keep the diameter $\mathcal {H}$ of the coarse grid as small as possible, due to its quadratic influence on the condition number.
